# Supplementary material for: Spatial Variation in Stream-Water Bacterial Communities Across Sites Differing in Wild Chinese Giant Salamander Detection Frequency
Source: Biology (Basel). 2026 Sep 18;15(18):1647. doi: 10.3390/biology15181647 (PMC13604818; doi:10.3390/biology15181647)
Supplement: Supplementary file 1 [file biology-15-01647-s001.zip › Data Related to “Spatial Variation in Stream-Water Bacterial Communities across Sites Differing in Wild Chinese Giant Salamander Detection Frequency”/Microbial Data/result/07.CorrelationAnalysis/07.CorrelationAnalysis_ReadMe.pdf]

扩增子结果文件补充说明(07)

2023 年 8 月 28 日

07.CorrelationAnalysis 【关联分析】

1. micropita 【microPITA筛选具有代表性的样本】

microPITA 软件有5 种筛选方式可供选择：包括 3 种无监督的方法和 2 种有监督的方法，具体方法描述如下，详细说明参考 micropitaReadme.pdf。

种类	筛选方法	描述
无监督（无分组）	Diverse	筛选α多样性最大的样品
	Representative	根据β多样性筛选反映整体差异的代表性样品
	Extreme	根据β多样性筛选最大差异的样品
有监督（有分组）	Distinct	筛选各分组之间β多样性筛选各分组之间β多样性
	Discriminant	筛选各分组处于中心位置的样品

1.1 otu_abundance.xls 【输入数据】

1.2 group.xls 【分组信息】

1.3 Discriminant/Representative/Extreme/Distinct/Discriminant【5种方法筛选样本结果】

1.3.1 diverse.txt/representative.txt/extreme.txt/distinct.txt/discriminant.txt【discriminant 方法筛 选样本结果】

1.3.2 PCoA 【PCoA 分析结果】

1.3.2.1PCoA.{xls,pdf,png} 【 PCoA 作图数据及未标样品名称的 PCoA 图】

1.3.2.2PCoA_label.{pdf,png}【标有样品名称的 PCoA 图】

1.3.2.3 PCoA_circle.{pdf,png} 【未标样本名带有置信椭圆的 PCoA 图】

1.3.2.4PCoA_circleLabel.{pdf,png} 【标有样本名带有置信椭圆的 PCoA 图】

2. network 【 network 分析结果】

2.1 group1 【组间 network 分析结果】

2.1.1 */dot/genus.{png,svg} 【network结果图形展示】

2.1.2 */dot/genus.spearman.index.list 【network 网页图绘制节点文件】

2.1.3 */dot/node.xls 【network 网页图绘制边文件】

2.1.4 */dot/igraph.calculate.txt 【network图中的关键参数展示】

列数	参数	含义
1	ND(Network diameter)	网络图直径
2	MD(modularity)	模块
3	CC(Clustering coefficient)	聚类系数
4	GD(graph density)	网络图密度
5	AD(Average degree)	平均连接度
6	APL(average.path.length)	平均路径长度

3. network3D 【 network3D 分析结果】

3.1 cytoplot.csv 【network3D 本地化Cytoscape绘制网络图数据】

3.2 edge.xls 【network3D 网页图绘制节点文件】

3.3 node.xls 【network3D 网页图绘制边文件】

3.4 netfig 【 network3D 网页展示文件】
